# Unraveling the multifaceted roles of SPL transcription factors in leaf development

**DOI:** 10.3389/fpls.2025.1696036

**Published:** 2025-11-19

**Authors:** Faujiah Nurhasanah Ritonga, Yiran Xu, Bing Cui, Xingfu Liu, Jianwei Gao, Jingjuan Li

**Affiliations:** 1 Shandong Key Laboratory of Bulk Open-field Vegetable Breeding, Ministry of Agriculture and Rural Affairs Key Laboratory of Huang Huai Protected Horticulture Engineering, Institute of Vegetables, Shandong Academy of Agricultural Sciences, Jinan, China; 2 Faculty of Forestry, Universitas Sumatera Utara, Medan, Indonesia; 3 College of Horticulture Science and Engineering, Shandong Agricultural University, Taian, China

**Keywords:** leaf development, miR156, plant architecture, SPL transcription factors, stress response

## Abstract

SQUAMOSA Promoter-Binding Protein-Like (SPL) transcription factors are a plant-specific family of regulatory proteins defined by a conserved SBP DNA-binding domain. They play essential roles in plant growth and development, coordinating processes such as the transition from juvenile to adult phase, branching, flowering time, and organ morphogenesis. SPL activity is tightly regulated by the miR156/157 pathway, forming a critical developmental module that integrates intrinsic and environmental cues. Recent research has expanded their known functions beyond development, revealing that SPLs also contribute to plant responses to abiotic stresses such as drought, salinity, nutrient deficiency, and temperature extremes, as well as biotic stresses including pathogen attack. Functional genomics studies across diverse species, including Arabidopsis, rice, maize, and forest trees, have uncovered both conserved and species-specific roles, emphasizing SPLs as key regulatory hubs in plant adaptation and productivity. This review summarizes advances in understanding SPL gene evolution, regulatory mechanisms, and interaction networks, with a focus on their relevance to plant architecture, leaf development, stress tolerance and crop improvement. Future applications of SPL research, particularly through gene editing, molecular breeding, and biotechnological innovations, present opportunities to optimize plant architecture, enhance resilience, and support sustainable agriculture and forestry in the face of climate change.

## 1 Introduction

Plant growth and development are governed by complex regulatory networks, with transcription factors (TFs) functioning as key modulators of gene expression in response to both intrinsic developmental cues and environmental conditions (Khoso et al., 2022). Acting as molecular switches, TFs influence a wide range of physiological processes including meristem activity, organ initiation, phase transitions, and responses to abiotic stress (Huang et al., 2021; Chopy et al., 2023; John et al., 2024). Their regulatory versatility makes them central to the coordination of plant form, adaptability, and productivity (Ritonga et al., 2021).

Among these TFs, the SQUAMOSA Promoter-Binding Protein-Like (SPL) family constitutes a lineage-specific group unique to the plant kingdom (Chen et al., 2010). SPL proteins possess a conserved SQUAMOSA Promoter-Binding Protein (SBP) domain that interacts with GTAC motifs in the promoters of target genes (Zhang et al., 2022). First identified in *Antirrhinum majus* and later in *Arabidopsis thaliana*, SPLs have been studied in diverse species, including *Oryza sativa*, *A. thaliana*, and *Triticum aestivum* (Xie et al., 2006; Wang et al., 2009; Li et al., 2022). These TFs are best known for regulating developmental phase transitions, flowering time, branching, and organ morphogenesis (Chen et al., 2010). Their expression is post-transcriptionally repressed by microRNAs, particularly miR156 and its paralog miR157, a mechanism that confers age-dependent control over SPL activity (Wang and Wang, 2015; Zhu et al., 2022). Functional studies have revealed that certain SPLs, such as SPL2, SPL5, and SPL16, exhibit partially redundant but distinct roles in shaping plant architecture through temporal and tissue-specific expression patterns (Cao et al., 2019; Sun et al., 2024; Zhang et al., 2024).

Leaf development represents a key facet of plant morphogenesis, determining photosynthetic capacity, plant architecture, and stress resilience. Traits such as leaf shape, curvature, angle, and size influence light interception, gas exchange, and developmental timing, and are therefore of agronomic importance (Ritonga et al., 2023; Nikolopoulos et al., 2024). Recent research has uncovered that SPLs function at multiple levels of leaf development, influencing processes such as juvenile-to-adult phase transitions, adaxial–abaxial polarity, and leaf blade curvature (Wang et al., 2021; Li et al., 2025a). For instance, HB34 regulate plant architecture in *Arabidopsis* by forming a regulatory module with miR157 and SPL10. HB34 directly represses miR157 and activates *AtSPL10*, establishing a feed-forward loop that influences branching and inflorescence structure in *Arabidopsis* leaves (Lee et al., 2022), while SPL9 contributes to freezing tolerance in *A. thaliana* by directly controlling the expression of the *AtCBF2* gene (Zhao et al., 2022b). Additionally, SPLs integrate hormonal signals such as cytokinin, gibberellin, and auxin, modulating growth plasticity under varying environmental conditions (Song et al., 2020).

In this review, we aim to integrate current knowledge of how SPL TFs regulate leaf development across plant species. We examine their molecular interactions with genetic and hormonal pathways, their roles in developmental transitions and morphogenesis, and their responses to environmental cues. We also discuss the potential applications of SPLs in crop improvement, particularly in optimizing leaf traits for enhanced light capture, improved canopy architecture, and increased stress tolerance. By linking fundamental discoveries from model systems with translational insights from crop research, we highlight the multifaceted role of SPLs in shaping plant form and performance.

## 2 Overview of SPL transcription factors

The SQUAMOSA Promoter-Binding Protein-Like (SPL) transcription factors constitute a plant-specific gene family initially discovered in *A. majus* due to their capacity to bind the promoter of the floral meristem identity gene SQUAMOSA (Preston and Hileman, 2010). Since their discovery, SPL genes have been characterized in various plant species, where they regulate a wide range of developmental processes, including phase transitions, flowering, organ morphogenesis, and responses to environmental cues (Chen et al., 2010; Song et al., 2020; Zhu et al., 2022). A defining feature of SPL proteins is the presence of the SBP (SQUAMOSA Promoter-Binding Protein) domain a highly conserved DNA-binding domain comprising around 76 to 80 amino acids, featuring two zinc finger motifs and a nuclear localization signal (NLS) (Li et al., 2020a). The SBP domain specifically binds GTAC core motifs in the promoter regions of target genes, thereby modulating gene expression programs critical for plant growth (Birkenbihl et al., 2005).

The SPL gene family exhibits variation in size across different plant species. *A. thaliana* contains 16 SPL genes, while *O. sativa* has 19, and *Zea mays* possesses up to 31 SPL members (Wu and Poethig, 2006; Xie et al., 2006; Mao et al., 2016). These numbers reflect lineage-specific expansions and gene duplication events, which have contributed to both functional redundancy and divergence among SPL family members. Phylogenetic analyses have grouped SPLs into several distinct clades based on sequence similarity and domain architecture, suggesting evolutionary specialization (Sun et al., 2021; He et al., 2022). Although some SPL genes share overlapping functions, others have acquired unique roles in tissue- or stage-specific development (Zhang et al., 2025) (**Table 1**).

**Table 1:** Overview of SPL genes and their regulatory roles in plant architecture across species.

SPL gene	Interacting gene(s)	Function	Interaction type	Species	Reference
*AtSPL10*	REVOLUTA (REV)	Promotes adaxial identity, regulates leaf curvature	Protein–protein interaction; direct target	*A. thaliana*	(Xu et al., 2025a)
*AtSPL2/9/13/15*	miR156/157 and AHL15/20	slow down plant ageing	miRNA–target regulation	*A. thaliana*	(Rahimi et al., 2022)
*AtSPL10*	miR156 and AGL79	Control plant architecture (narrow leaves)	miRNA–target regulation	*A. thaliana*	(Gao et al., 2017)
*BpSPL4/SPL9*	miR156	Regulates leaf and lateral branch development	miRNA–target	*Betula platyphylla*	(Yan et al., 2024)
*AtSPL10*	HB34 and miR157	Modify branching and inflorescence architecture	miRNA–target	*A. thaliana*	(Lee et al., 2022)
*GmSPL9*	*miR156b*	Alter soybean architecture	miRNA–target	*G. max*	(Bao et al., 2019)
*OsSPL14 (IPA1)*	miR156	Alters leaf angle and erectness through ideal plant architecture pathway	miRNA–target	*O. sativa*	(Jiao et al., 2010)
*BrpSPL9*	miR156	Regulates heading time and leaf folding in Chinese cabbage	miRNA–target	*B. rapa*	(Wang et al., 2014)

**Figure 1:**
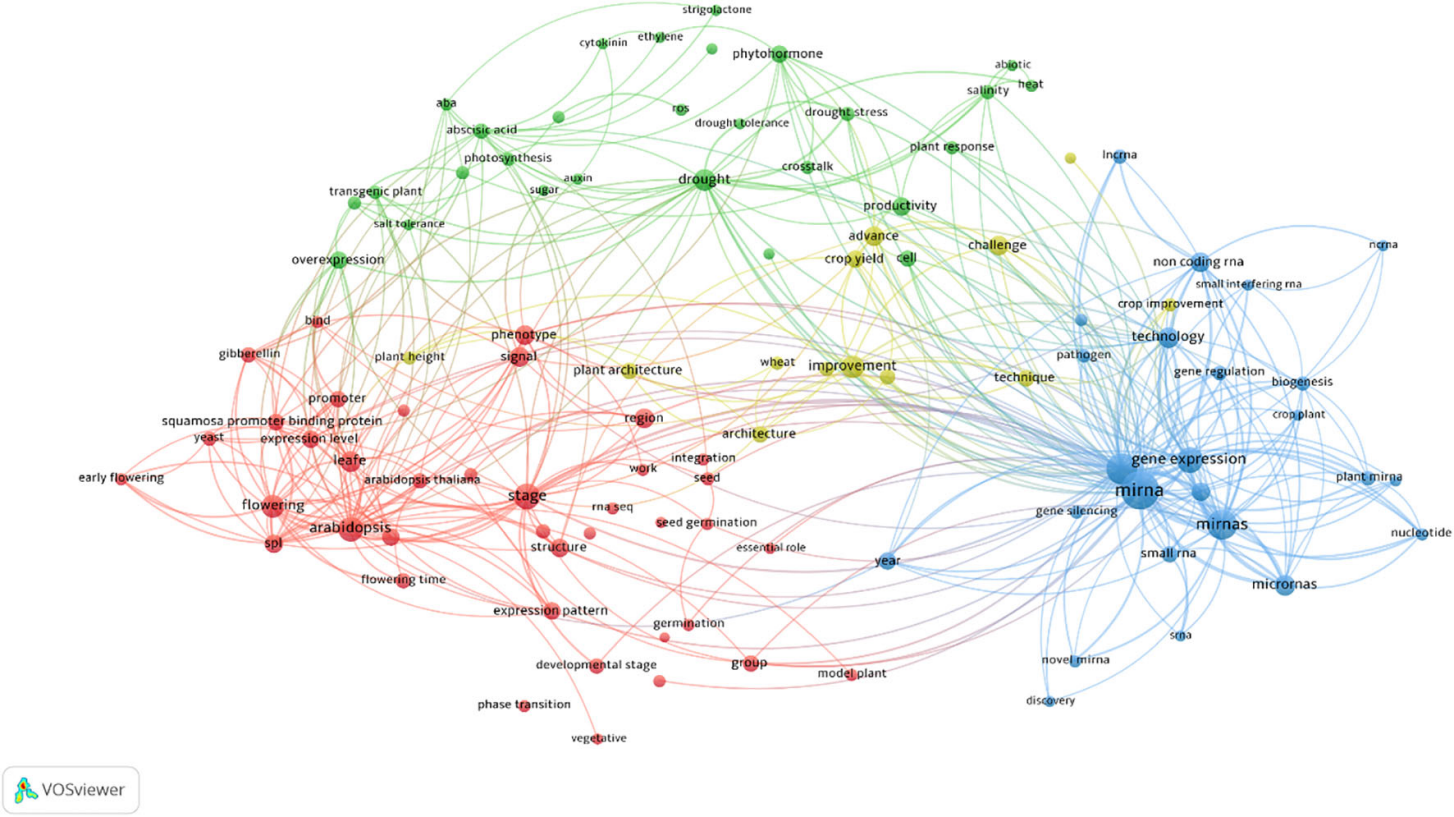
Visualization of research trends related to SPL genes in plants over the past five years using a term co-occurrence map of SPL studies using VOSviewer software version 1.620 (https://www.vosviewer.com/). The figure highlights miR156, plant architecture, abiotic stress, and hormonal regulation as major research themes closely associated with SPL studies. (accessed: 24 August 2025).

Post-transcriptional regulation plays a key role in SPL gene function, particularly through microRNAs such as miR156 and miR157 (Lee et al., 2022; Zhang et al., 2025). These conserved miRNAs target sites within the coding region or 3′ untranslated region (3′-UTR) of SPL transcripts, resulting in mRNA cleavage or translational repression (Zhu et al., 2022). In *Arabidopsis*, 10 out of 16 SPL genes are regulated by miR156, and similar patterns are observed in other species, including rice and maize (Wu and Poethig, 2006; Xie et al., 2006; Mao et al., 2016). The expression of miR156 is developmentally regulated high during early vegetative stages and declining as plants mature thus timing the activation of SPL genes that promote adult traits such as leaf complexity, shoot maturation, and floral induction (Song et al., 2020; Li et al., 2022). Functional studies have shown that specific SPLs, such as SPL9 and SPL15, act redundantly in controlling vegetative phase change. In contrast, others like SPL3, SPL4, and SPL5 specialize in promoting floral development, illustrating the functional divergence within this gene family (Wu and Poethig, 2006; Zhao et al., 2022b; Yan et al., 2024). In the past five years, research trends on SPLs in plants have been summarized in **Figure 1**. The figure highlights that miR156, plant architecture, abiotic stress, and hormones are key topics closely associated with SPL studies.

## 3 Regulation and functional integration of SPLs in leaf development

The expression of SPL genes is precisely regulated in both space and time to synchronize plant development. In general, SPL transcripts are low in juvenile tissues and accumulate progressively during vegetative growth, marking the transition to the adult phase. Spatially, different SPLs exhibit distinct expression patterns; some are preferentially expressed in shoot apices and young leaves, while others localize to reproductive meristems or vascular tissues. For instance, *AtSPL10* is primarily expressed in leaf primordia and midveins in *A. thaliana* (Xu et al., 2025a), where it influences leaf polarity and curvature. In contrast, *BrSPL9* exhibits broader expression in shoot and leaf tissues, affecting both phase change and morphogenesis in *Brassica rapa* (Wang et al., 2014).

The regulation of SPL expression is under tight developmental control, primarily through the age pathway mediated by miR156 (Jiao et al., 2010). During early vegetative growth, high levels of miR156 suppress SPL transcripts. As the plant ages, miR156 levels decline, allowing *OsSPL* expression to rise and promote adult traits such as leaf serration, curvature, and the initiation of reproductive development (**Table 2**). In addition to age, SPLs respond to hormonal and environmental cues (Song et al., 2023). In *Arabidopsis*, cytokinin has been shown to induce *AtSPL10* expression through ARR1, integrating hormonal signals with developmental timing (Barrera-Rojas et al., 2020). Other hormones such as auxin and gibberellins (GA) also influence SPL activity, either by modulating miR156 levels or through interaction with SPL targets, suggesting a complex network of regulatory feedback in Pyrus (Song et al., 2020).

**Table 2:** Overview of SPL genes and their roles in biotic and abiotic stress responses.

Environmental signal	SPL gene	Interacting gene(s)	Function	Interaction type	Species	Reference
Drought stress	*TaSPL6*	NA	Enhance sensitivity to drought stress	NA	*Triticum aestivum*	(Zhao et al., 2024)
	*EoSPL1-EoSPL19*	miR156	Diverse responses to abiotic stress	miRNA–target regulation	*Eremochloa ophiuroides*	(Kong et al., 2025)
Salt stress	*OsSPL1*	NA	Enhance sensitivity to exogenous abscisic acid (ABA), and decreased tolerance to salt and oxidative stress	NA	*O. sativa*	(Zhang et al., 2012)
	*AhSPL5*	ERF, WRKY, MYB, Dof, and microRNAs, like ahy-miR156	Enhance salt tolerance in transgenic Arabidopsis	miRNA–target regulation	*Arachis hypogaea*	(Sun et al., 2024)
Water stress	HaSPL	miR156	Broad involvement of HaSPLs in the response to flood and drought stresses	miRNA–target regulation	*Helianthus annuus*	(Jadhao et al., 2023)
Heat stress	*AtSPL1* or *AtSPL12*	ABAreceptorsPYR1/PYL1/PYL2/PYL4/PYL5/PYL8	Play a crucial role in the mechanisms of plant thermotolerance at the reproductive stage	NA	*A. thaliana*	(Chao et al., 2017)
Cold/freezing stress	*BvSPLs*	NA	Participate in the regulation of root expansion and sugar accumulation.	NA	*Beta vulgaris*	(Xue et al., 2024)
	*AtSPL9*	CBF2	control the expression of the CBF2 gene	NA	*A. thaliana*	(Zhao et al., 2022b)
Abiotic stresses	*CqSPLs*	NA	Play a critical role in quinoa development and in its response to various abiotic stresses	NA	*Chenopodium quinoa*	(Ren et al., 2022)
	*SbSPL7/9/10/12*	miR156/157	Upregulate in response to abiotic stress	miRNA–target regulation	*Scutellaria baicalensis*	(Wu et al., 2024)
	*AtSPL9*	miR156	Essential for activation of ABA responses	miRNA–target regulation	*Arabidopsis*	(Dong et al., 2021)
*Ciboria shiraiana* stress	*MaSPL8*	miR5658 and miR4221	integrate with phytohormone pathways	miRNA–target regulation	*Morus alba*	(Zheng et al., 2025)

**Figure 2:**
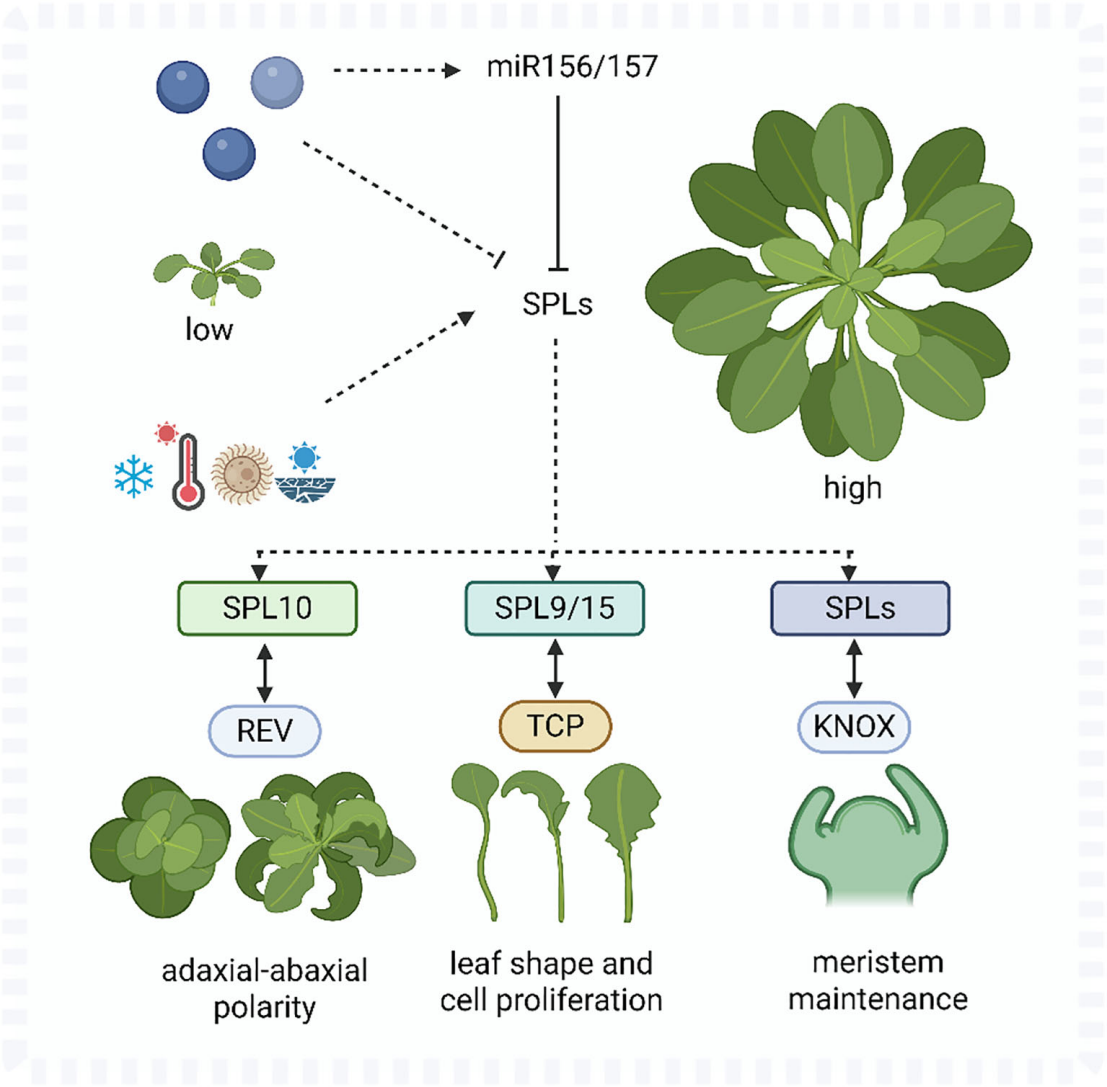
A schematic model of SPL gene regulation and functional integration in leaf development. SPL expression is modulated by miR156, hormones (cytokinin, auxin, and GA), and transcription factors (REV, TCP, and KNOX), thereby coordinating developmental phase transitions, leaf polarity, and morphogenesis. The blue spheres indicating that they represent other regulatory genes that can either repress or enhance the expression of miR156/157.

SPLs function through both direct and indirect interactions with key transcriptional regulators involved in leaf patterning (Chen et al., 2010). For example, *AtSPL10* modulates leaf curvature by interacting with the HD-ZIP III transcription factor *REVOLUTA* (REV), forming a module that coordinates adaxial-abaxial polarity (Xu et al., 2025a). *AtSPL9* and *AtSPL15* are known to regulate or interact with TCP TFs, which are critical in controlling leaf shape and cell senescence (Hyun et al., 2016). Furthermore, *AtSPLs* exhibit antagonistic or synergistic relationships with *AtKNOX* genes, which play roles in meristem maintenance and compound leaf development (Roth et al., 2018). Hormonal pathways converge on these interactions. Cytokinin promotes *AtSPL* expression via ARR1 (Barrera-Rojas et al., 2020), while auxin may counteract SPL-mediated processes during organ initiation (Liu et al., 2019). Together, these cross-regulatory interactions position SPLs as key integrators of developmental timing, environmental adaptation, and hormonal signaling in shaping leaf morphology (**Figure 2**).

## 4 SPLs in leaf curvature and morphogenesis

Leaf curvature is a fundamental architectural trait that determines the efficiency of light interception, gas exchange, and overall plant productivity (Tabusam et al., 2023). It results from differential cell growth along the adaxial (upper) and abaxial (lower) surfaces of the leaf, often governed by a complex regulatory network involving polarity genes, hormonal signaling, and transcriptional programs (Tang et al., 2009). In model species such as *A. thaliana*, recent studies have identified SPL TFs, particularly *AtSPL10*, as key regulators of leaf curvature, acting through both genetic and hormonal modules to control this finely tuned process (Xu et al., 2025a).

A pivotal study by Xu et al. (2025a) demonstrated that SPL10 interacts directly with REVOLUTA (REV), an HD-ZIP III transcription factor known to promote adaxial leaf identity. Through physical interaction and co-expression, *AtSPL10* and REV form a regulatory module that coordinates adaxial–abaxial polarity and defines curvature outcomes. Overexpression of *AtSPL10* in *Arabidopsis* resulted in severely curled leaves, whereas loss-of-function *spl10* mutants partially rescued the curled-leaf phenotype in *rev* mutants, highlighting functional convergence. This genetic interaction mirrors earlier findings on HD-ZIP III (REV) and KANADI pathways, suggesting that SPLs operate within established polarity networks (Emery et al., 2003; Husbands et al., 2009). In addition, ZF-HD transcription factors, especially HB34, regulate shoot architecture in *Arabidopsis* by repressing miR157 and promoting the expression of its target gene, SPL10 (Lee et al., 2022).

Beyond its direct regulation of REVOLUTA (REV), *BrSPL10* may influence broader leaf polarity pathways in Chinese cabbage (Xu et al., 2025a). Although SPL10 influences leaf morphology and polarity through regulatory modules involving REVOLUTA (REV), there is no evidence that SPL10 directly regulates abaxial determinant genes such as KANADI or YABBY. Any observed effects on leaf polarity are more likely mediated indirectly through REV- or BOP-dependent pathways (Gao et al., 2018; Hu et al., 2023).

The phenotypic consequences of SPL10 manipulation are striking: while overexpression results in upward-curled, narrow leaves, *spl10* mutants display more flattened, expanded leaf blades. This phenotype is partially rescued in *rev* sp*l10* double mutants, confirming the antagonistic yet cooperative function of *AtSPL10* and REV (Xu et al., 2025b). These findings parallel observations in other SPLs such as SPL9, which also contribute to morphogenetic traits, though with less direct influence on curvature. Comparative studies across species remain limited, but the regulatory logic appears conserved; for instance, *BrpSPL9* in *B. rapa* affects heading time and leaf folding, possibly through similar polarity and growth control pathways (Wang et al., 2014). In addition, AGAMOUS-like MADS box protein 79 (AGL79) regulates plant development in a dose-dependent manner, affecting leaf morphology, shoot branching, and flowering. *AtSPL10* directly activates it and acts downstream of the miR156/SPL10 module to influence lateral root growth (Gao et al., 2017).

Understanding the role of SPLs in leaf curvature has important implications for agriculture (Wang et al., 2022). In crops like rice and maize, optimal leaf angle, a trait closely tied to curvature and blade architecture, is critical for maximizing light interception and yield under dense planting. While most SPL research has been centered on *Arabidopsis*, translational insights are emerging (Li et al., 2020b). For example, *OsSPL14* (IPA1), a gene regulated by *OsmiR156*, plays a crucial role in shaping the ideal plant architecture in rice by reducing tiller quantity, enhancing lodging resistance, and increasing grain yield (Jiao et al., 2010). Bridging these studies with knowledge from *Arabidopsis* SPL10-REV systems offers potential routes for engineering ideotype leaves with favorable curvature and angles for enhanced photosynthetic efficiency and crop improvement.

## 5 SPLs in leaf senescence and maturation

Leaf senescence is the final developmental stage of a leaf’s lifecycle, marked by coordinated processes such as chlorophyll degradation, nutrient remobilization, and programmed cell death (Guo et al., 2021; Ritonga et al., 2023). It is tightly regulated by both internal developmental cues and external environmental factors. Among the internal regulators, the miR156–SPL module has emerged as a central age-dependent mechanism that coordinates the timing of leaf maturation and senescence (Xu et al., 2016). In young plants, high levels of miR156 suppress the expression of its target SPL genes, maintaining juvenile traits and delaying aging. As the plant matures, miR156 levels decline, while miR172 levels increase, leading to the gradual activation of SPL transcription factors that promote adult-phase characteristics, including leaf ageing (Vander Schoor et al., 2022).

SPL9 is a key transcription factor involved in developmental phase change via the miR156–SPL module. To date, no studies have explicitly shown that SPL9 directly regulates ORE1, SAG29, or chlorophyll biosynthesis genes (Seo et al., 2011; Rauf et al., 2013). These senescence pathways may operate independently or downstream of other regulators. In contrast, SPL13 has been implicated in age-related developmental transitions, although its role in leaf senescence appears to be less pronounced than that of SPL9. Notably, recent findings suggest that *AtSPL13* orthologs can respond to hormonal cues such as abscisic acid (ABA) and ethylene, potentially linking hormonal signals with age-related gene expression (Song et al., 2023).

**Figure 3:**
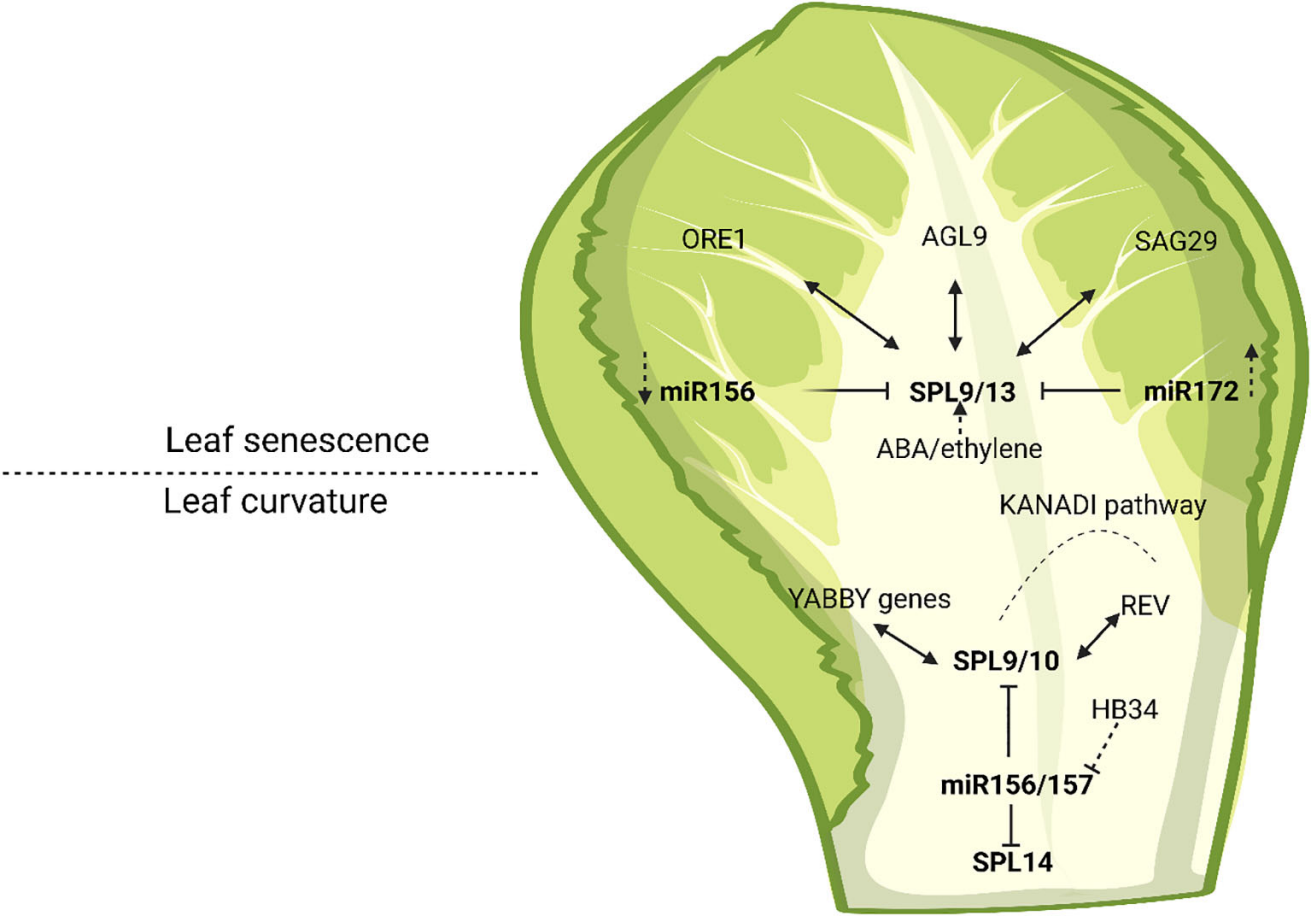
Network of genes associated with SPL transcription factors and the miR156/miR172 regulatory modules in controlling leaf curvature and senescence. The figure illustrates how SPLs integrate age-related miRNA signaling with hormonal and transcriptional pathways to modulate leaf polarity, morphogenesis, and aging processes.

Although direct evidence linking SPLs to nutrient remobilization is limited, SPL transcription factors, particularly SPL9 have been shown to regulate age-dependent developmental transitions and influence the expression of senescence-associated genes. These include genes involved in chlorophyll degradation and leaf maturation, highlighting their role in the timing and progression of leaf senescence. The upregulation of SPLs during later developmental stages reflects their function as phase identity markers, bridging the transition from juvenile to adult stages and reproductive competence. Collectively, current findings support SPLs, especially those regulated by the miR156 pathway, as essential components in the genetic network that integrates age cues with transcriptional regulation of senescence (**Figure 3**) (Xu et al., 2016).

## 6 Crosstalk between SPLs and environmental signals

Plants constantly adjust their development in response to changing environmental conditions, and TFs, such as SPLs, serve as critical hubs that integrate internal genetic programs with external signals (Ritonga et al., 2021). Recent studies have shown that SPL gene expression and activity can be modulated by environmental factors, including light intensity, temperature fluctuations, and abiotic stressors such as drought, salinity, and nutrient limitation (Zhao et al., 2022b; Jing et al., 2025). Light-regulated developmental transitions, for instance, are partly mediated by changes in miR156 expression, which in turn affects the timing of SPL gene activation. Under extended photoperiods or high light conditions, a reduction in miR156 leads to increased SPL activity and the advancement of developmental events, such as leaf expansion and senescence (Cao et al., 2023).

Temperature extremes and abiotic stress also alter the function of SPL in leaves. Several SPLs, including SPL9 and SPL13, have been reported to participate in stress adaptation mechanisms, often through downstream targets involved in hormone signaling, redox regulation, and cell wall modification (LaFountain and Yuan, 2021; Ma et al., 2021; Zhao et al., 2022b). For example, under aluminum stress, SPL13 expression increases while miR156 is suppressed in alfalfa roots. Overexpression of miR156 leads to higher Al accumulation, membrane damage, and nutrient loss, whereas increased SPL13 enhances root length and Al tolerance. Transcriptome and ChIP-seq analyses revealed that SPL13 regulates genes involved in Al response, including transporters, transcription factors, and cell wall-associated proteins (Allam et al., 2025). Additionally, SPLs may indirectly mediate tolerance by modulating leaf structure and growth, enabling adjustments in leaf size, angle, or curvature to reduce water loss or optimize light capture under adverse conditions (Li et al., 2024; Bu et al., 2025). Furthermore, overexpression of *BpmiR156* resulted in the transcriptional downregulation of *BpSPL4* and *BpSPL9*, accompanied by differential expression of hormone-related genes involved in auxin and cytokinin biosynthesis, including *BpARR3*, *BpARR11*, and *BpmiR172* (Yan et al., 2024).

At the molecular level, epigenetic and transcriptional reprogramming play a role in linking stress with SPL regulation. Environmental stress can affect histone modifications and DNA methylation at the MIR156 locus or SPL promoters, thereby shifting the expression thresholds of these genes reversibly (Bu et al., 2025). Moreover, SPLs themselves may be subject to transcriptional repression or activation by stress-induced transcription factors, such as DREB or WRKY family members, which are known to bind to the promoter regions of development-related genes (Zhao et al., 2022b). These layers of regulation enable a flexible and context-dependent role for SPLs in tuning leaf growth and developmental timing under environmental stress, underscoring their importance in shaping both plant form and resilience (Zheng et al., 2019; Jerome Jeyakumar et al., 2020).

## 7 SPLs in crop leaf development

While much of our mechanistic understanding of SPL TFs stems from studies in *A. thaliana*, recent research has expanded to include several economically important crops including rice, maize, wheat, barley and sorghum, revealing both conserved and specialized roles for SPLs in regulating leaf development, plant architecture, and yield-related traits (Liu et al., 2019; Giaume and Fornara, 2021; He et al., 2024; Zhong et al., 2024). In rice, *SPL14* regulates leaf angle and tiller number, contributing to higher planting density and improved yield (Jiao et al., 2010). However, in another study, it was confirmed that *OsSPL14* enhances rice grain appearance by reducing chalkiness through direct activation of Wx and PDIL1-1, key genes involved in starch and protein regulation. It also interacts with NF-Y transcription factors to promote their expression. Loss of OsSPL14 impairs endosperm development, highlighting its crucial role in improving grain quality (Li et al., 2025b). In maize (*Zea mays*), SPL genes such as *ZmSPL12* have been linked to plant height, leaf width, and photosynthetic efficiency (Zhao et al., 2022a). In wheat (*Triticum aestivum*), SPL family members are involved in flag leaf morphology, influencing grain filling and biomass accumulation (Liu et al., 2019). Notably, in Chinese cabbage (*Brassica rapa* ssp. *pekinensis*), *BrpSPL9* has been shown to regulate the earliness of heading time by affecting leaf incurvature, a key trait for head formation (Wang et al., 2014).

Given their central roles in leaf architecture, manipulation of SPL genes has emerged as a promising strategy for enhancing crop traits. CRISPR/Cas9-mediated modification of SPL genes has been successfully applied in crops such as tomato and soybean. In tomato, editing the *SPL-CNR* gene impaired fruit ripening, ethylene production, carotenoid accumulation, and volatile synthesis, confirming SPL-CNR’s central role in ripening regulation (Do et al., 2024). In soybean, simultaneous mutation of multiple *GmSPL9* genes using CRISPR/Cas9 led to changes in node and branch number, demonstrating the potential of SPL gene editing to improve plant architecture and yield-related traits (Bao et al., 2019). In Chinese cabbage, altering *BrpSPL9* expression can control heading time and leaf folding, which are crucial for market quality (Wang et al., 2014). Unfortunately, there is no published evidence of CRISPR/Cas9-mediated modification of SPL family genes in Chinese cabbage.

The biotechnological potential of SPLs extends beyond trait modification to the development of climate-resilient soybean. By targeting SPLs that interface with hormonal and environmental pathways, breeders can develop plants that adapt their leaf morphology to stress conditions such as drought or high planting density. Genome editing technologies, such as CRISPR/Cas9, offer precise tools to manipulate specific SPL loci without introducing foreign genes, thereby enhancing the acceptance of soybean in regulatory frameworks (Bao et al., 2019). As research continues to uncover the molecular targets and networks controlled by SPLs, these transcription factors emerge as valuable levers in designing next-generation rice, barley, wheat, sorghum with optimized canopy structure, enhanced photosynthetic efficiency, and improved yield potential.

## 8 Challenges and future perspectives

Despite significant advances in understanding the roles of SPL transcription factors, several challenges remain that limit the full exploitation of their potential in both basic research and crop improvement. Most current studies focus on a few well-characterized SPLs (e.g., SPL3, SPL9, SPL10, SPL14) in model plants like Arabidopsis and rice, leaving the functions of many other family members unexplored, especially in non-model and economically important species (Bu et al., 2025). Additionally, the phenotypic redundancy among SPL paralogs often masks loss-of-function effects, complicating the functional dissection (Preston and Hileman, 2010; 2013). Moreover, the molecular mechanisms linking SPL activity to cellular and tissue-level changes in leaf morphology are still incomplete, particularly regarding downstream targets, spatial specificity, and cross-regulatory feedback (Li et al., 2024).

To overcome these limitations, future research will benefit from integrated multi-omics and systems biology approaches, including transcriptomics, proteomics, epigenomics, and metabolomics (Tyagi et al., 2022). Such strategies can unravel the broader regulatory networks in which SPLs are embedded and identify dynamic changes during leaf development or in response to environmental conditions. Computational modeling, gene regulatory network mapping, and cell-type-specific expression profiling will also enhance our ability to predict SPL functions under diverse developmental and environmental conditions (Van den Broeck et al., 2020; Saint-André, 2021; Fu et al., 2024). These comprehensive approaches are especially important for translating findings from Arabidopsis to crops, where environmental variability and complex traits require a systems-level understanding.

The emergence of precise genome editing tools such as CRISPR/Cas9 has opened new avenues for SPL-based breeding strategies (Razzaq et al., 2021). By targeting individual SPL genes or their regulatory elements, such as miR156-binding sites or promoter regions, researchers can modulate leaf architecture traits in a controlled manner. Looking ahead, emerging research into non-coding RNAs, including long non-coding RNAs (lncRNAs) and circular RNAs (Liu et al., 2015; Zhang and Dai, 2022), suggests new layers of post-transcriptional SPL regulation that remain largely unexplored. Furthermore, post-translational modifications of SPL proteins, such as phosphorylation, ubiquitination, or interaction with chromatin remodelers, may fine-tune their stability and activity in a context-dependent manner (Li et al., 2025c). Expanding our knowledge in these areas will be critical for unlocking the full potential of SPLs as master regulators of leaf development and stress adaptation in crops.

## 9 Conclusion and future perspective

SQUAMOSA Promoter-Binding Protein-Like (SPL) transcription factors are central regulators of plant development, orchestrating genetic, hormonal, and environmental signals to shape plant architecture, flowering, and stress responses. Advances in functional genomics and molecular genetics have demonstrated the highly conserved yet functionally diverse roles of these fields across species, influencing critical traits such as vegetative-to-reproductive phase transitions, reproductive development, and tolerance to abiotic and biotic stresses. However, significant knowledge gaps remain, particularly in understanding species-specific SPL regulatory networks, their interactions with other transcription factors, and their evolutionary diversification in both crop and forestry species. Future research integrating high-resolution transcriptomics, advanced gene-editing technologies, and comparative genomics will be crucial to unlock the full potential of SPL genes in plant breeding. Harnessing SPL functions through targeted genetic engineering presents promising opportunities to optimize plant architecture, increase yield, and enhance resilience to climate change. By bridging molecular discoveries with applied breeding strategies and biotechnological innovation, SPL transcription factors can be transformed from fundamental research targets into practical tools for sustainable agriculture and forestry.
